# Parental Education and Left Lateral Orbitofrontal Cortical Activity during N-Back Task: An fMRI Study of American Adolescents

**DOI:** 10.3390/brainsci11030401

**Published:** 2021-03-22

**Authors:** Shervin Assari, Shanika Boyce, Mohammed Saqib, Mohsen Bazargan, Cleopatra H. Caldwell

**Affiliations:** 1 Department of Urban Public Health, Charles R Drew University of Medicine and Science, Los Angeles, CA 90059, USA; Mohsenbazargan@cdrewu.edu; 2 Department of Family Medicine, Charles R Drew University of Medicine and Science, Los Angeles, CA 90059, USA; 3 Department of Pediatrics, Charles R Drew University of Medicine and Science, Los Angeles, CA 90059, USA; ShanikaBoyce@cdrewu.edu (S.B.); cleoc@umich.edu (C.H.C.); 4 Department of Health Behavior and Health Education, School of Public Health, University of Michigan, Ann Arbor, MI 48104, USA; saqimoha@umich.edu; 5 Department of Family Medicine, University of California Los Angeles, Los Angeles, CA 90095, USA; 6 Center for Research on Ethnicity, Culture, and Health (CRECH), School of Public Health, University of Michigan, Ann Arbor, MI 48104, USA

**Keywords:** population groups, socioeconomic factors, adolescents, brain development, fMRI, cognitive, N-Back, memory, learning, orbitofrontal cortex

## Abstract

*Introduction.* The Orbitofrontal Cortex (OFC) is a cortical structure that has implications in cognition, memory, reward anticipation, outcome evaluation, decision making, and learning. As such, OFC activity correlates with these cognitive brain abilities. Despite research suggesting race and socioeconomic status (SES) indicators such as parental education may be associated with OFC activity, limited knowledge exists on multiplicative effects of race and parental education on OFC activity and associated cognitive ability. *Purpose.* Using functional brain imaging data from the Adolescent Brain Cognitive Development (ABCD) study, we tested the multiplicative effects of race and parental education on left lateral OFC activity during an N-Back task. In our study, we used a sociological rather than biological theory that conceptualizes race and SES as proxies of access to the opportunity structure and exposure to social adversities rather than innate and non-modifiable brain differences. We explored racial variation in the effect of parental educational attainment, a primary indicator of SES, on left lateral OFC activity during an N-Back task between Black and White 9–10 years old adolescents. *Methods.* The ABCD study is a national, landmark, multi-center brain imaging investigation of American adolescents. The total sample was 4290 9–10 years old Black or White adolescents. The independent variables were SES indicators, namely family income, parental education, and neighborhood income. The primary outcome was the average beta weight for N-Back (2 back versus 0 back contrast) in ASEG ROI left OFC activity, measured by functional Magnetic Resonance Imaging (fMRI) during an N-Back task. Ethnicity, age, sex, subjective SES, and family structure were the study covariates. For data analysis, we used linear regression models. *Results.* In White but not Black adolescents, parental education was associated with higher left lateral OFC activity during the N-Back task. In the pooled sample, we found a significant interaction between race and parental education on the outcome, suggesting that high parental education is associated with a larger increase in left OFC activity of White than Black adolescents. *Conclusions.* For American adolescents, race and SES jointly influence left lateral OFC activity correlated with cognition, memory, decision making, and learning. Given the central role of left lateral OFC activity in learning and memory, our finding calls for additional research on contextual factors that reduce the gain of SES for Black adolescents. Cognitive inequalities are not merely due to the additive effects of race and SES but also its multiplicative effects.

## 1. Introduction

Given that socioeconomic status (SES) indicators such as parental education [1] are closely associated with exposure to chronic stress [2] and social adversities, and given that stress and adversities jeopardize adolescents’ brain development [3,4,5,6], it should be no surprise that there is a connection between SES indicators such as parental education and adolescents brain development [7,8,9,10,11,12,13,14]. Adolescents from low and high SES experience vastly different social and economic adversity levels, thus showing considerable brain function changes [15,16,17]. As a result of poor brain development, adolescents from low SES families become at risk of undesired cognitive, emotional, and behavioral outcomes such as poor school performance [18], depression [19], anxiety [20,21], antisocial behaviors [22], aggression [23], and sexual initiation [24,25,26], as well as the use of tobacco [27,28], alcohol [29,30], and other drugs [31].

The effects of SES and associated stress are not specific to a particular brain region, but their effects are shown for multiple brain regions, including the amygdala [15,32,33], hippocampus [34,35,36], as well as the cerebral cortex. However, due to the scarcity of research on SES’s effects on brain regions, our understanding of brain regions that are affected by SES is inconsistent.

Among various brain regions and structures that carry the effect of SES is the Orbitofrontal Cortex (OFC), a cortical structure with major implications in cognition, memory, decision making, and learning. As such, OFC activity correlates with such cognitive brain abilities [37,38,39,40]. The OFC has been shown to be affected by stress and SES [41]. Individuals with low performance or shrinkage of the OFC may show poor learning ability and memory. Several studies have shown that race, SES, and stress impact OFC. Altered OFC structure and function are also shown to be a part of dementia [42], Alzheimer’s disease [43,44], psychosis [45,46], post-traumatic stress disorder (PTSD) [47,48], depression [49,50,51], and drug use [52,53,54].

Most of the existing literature on the link between SES and adolescents’ brain development has focused on various aspects of emotion regulation rather than domains of memory and cognition [15,32,33,55]. Similarly, most of the existing research on the effects of stress and SES is limited to amygdala structure and function [15,32,33,55]. For example, Javanbakht et al. have documented the effects of household SES and childhood stress on amygdala response to threatening stimuli [15,32,33]. However, less is known on the effect of SES and environment on the OFC [41,56].

Accurate knowledge regarding the nature of the undesired effect of low SES on brain development and function will help us better understand why low SES adolescents report worse developmental outcomes [57], school performance [57], mental health [58], emotion regulation [59,60], aggression [61], and substance use [58,62]. Such research-based knowledge on how SES operates as a social determinant of adolescents’ brain development and function is core for breaking the vicious cycle between low family SES and poor child developmental outcomes across multiple emotional and behavioral domains.

Theoretically, the scarcity hypothesis explains why and how SES deteriorates healthy adolescents’ brain development. According to this theoretical framework, low SES reflects the scarcity of resources that are essential for adolescents’ brain development. In this view, food and home insecurity increase the risk for poor child development. As such, poor access to resources that are buffers against poor developmental outcomes is one of the many mechanisms that may explain the link between low SES and poor brain development [63]. Low family SES is also a proxy of poor parenting [64,65,66,67,68] and high parental risk behaviors [69,70] that can put child brain development in jeopardy [71]. Secondary to these cumulative risks, adolescents from low SES families remain at high level of risk of psychopathologies [72,73,74], problem behaviors [75,76,77,78,79,80,81], and poor school performance [82,83,84].

Multiple reasons suggest the association between SES and race/ethnicity are complex and interactive. First, race/ethnicity and SES have a major overlapping distribution [85,86]. Low SES may even mediate (explain) the racial and ethnic disparities in adolescents’ brain development [87]. In addition, SES may have differential impacts on adolescent brain development across diverse racial and ethnic groups [55]. One study suggested that family income has stronger effects on brain function for the most disadvantaged than the least disadvantaged groups in the society [55].

In contrast, according to the Minorities’ Diminished Returns (MDRs) framework [88], racial and ethnic minorities show weaker associations between SES and outcomes [89,90]. In several studies in children, youth, adults, and older adults, family SES shows weaker effects for Blacks than Whites [87,91,92,93,94,95]. As a result of MDRs, while White youth from high SES backgrounds show the lowest level of risk, Black adolescents remain at high risk regardless of SES, a pattern similarly relevant to behavioral, developmental, and health outcomes [89,96]. These patterns are shown across emotional, behavioral, and cognitive domains such as high-risk behaviors [96], aggression [96] and tobacco use [97], anxiety [98], depression [99], poor health [87], chronic disease, obesity, poor school attachment, impulsivity, and poor school performance [83]. These indicate a novel mechanism of health inequalities which is systematically overlooked by researchers and policymakers and suggest health disparities are not just due to lack of access to SES but also societal inequalities that slow, hinder, and block the process of translation of an SES resource (e.g., parental education) to an outcome (e.g., youth brain development).

### Aims

To understand the social patterning of American adolescents’ brain development, we conducted this study with two aims: First, to study the effect of SES on left lateral OFC activity measured during an N-Back task. Using the Adolescent Brain Cognitive Development (ABCD) data, which are from the state-of-the-art study of adolescents’ brain development [100,101,102,103,104,105,106,107,108,109,110,111,112,113], we hypothesized that high parental education, as a major SES indicator, would be associated with a higher left lateral OFC activity. We also hypothesized that when comparing White and Black adolescents, the positive association between SES (parental education) and the left lateral OFC activity measured during an N-Back task would be weaker in Black than White adolescents, in line with the MDRs framework [88,96].

## 2. Methods

### 2.1. Study Design

We conducted a secondary analysis of the data from the Adolescent Brain Cognitive Development (ABCD) study [102,103,109,114,115]. This study applied a cross-sectional design and only used wave one of the ABCD data [100,101,102,103,104,105,106,107,108,109,110,111,112,113]. ABCD study is the largest brain imaging studies of adolescents in the US [109,116].

### 2.2. Sample and Sampling

Participants were recruited from school systems in 21 study sites, which were distributed across multiple states. The recruitment was limited to 9–10-year-old children. To increase the generalizability of the sample, schools were selected based on their distribution of race, ethnicity, SES, sex, and urbanicity. For more information, please consult a fully detailed description of the ABCD sample and sampling [112]. The current analysis was performed in 4290 9–10-year-old White or Black adolescents. Participants were included in this analysis if they had complete data on all study variables.

### 2.3. Variables

The study variables included demographic factors (age and sex), SES indicators (parental educational attainment, subjective SES), and left lateral OFC activity (mean beta weight for N-Back run 1 2 back conditions in APARC ROI left lateral orbitofrontal: tfmri_nback_r1_349). Left lateral OFC activity was measured using a task-based functional MRI measure during N-Back. Details of the procedures for harmonization of the fMRIs and imaging are explained elsewhere [102].

#### 2.3.1. Outcome

The outcome was left lateral OFC activity measured as mean beta weight for N-Back run 1 2 back conditions in APARC ROI left lateral orbitofrontal: tfmri_nback_r1_349. We selected the left lateral OFC because it is shown to be impacted by poverty, trauma, and adversity [56,117,118,119,120].

#### 2.3.2. Moderator

*Race.* Race was self-identified and treated as a dichotomous variable: Black = 1, White = 0 (reference group).

#### 2.3.3. Independent Variables

*Parental Educational Attainment.* Participants were asked, “What is the highest grade or level of school you have completed or the highest degree you have received?” Responses ranged from 0 (Never attended/Kindergarten) to 21 (doctoral degree). This variable ranged from 1 to 21.

*Financial Status.* This study measured financial status using the following seven items: “In the past 12 months, has there been a time when you and your immediate family experienced any of the following:” (1)“Needed food but couldn’t afford to buy it or couldn’t afford to go out to get it?“, (2) “Were without telephone service because you could not afford it?“ (3)“ Didn’t pay the full amount of the rent or mortgage because you could not afford it?“, (4) “Were evicted from your home for not paying the rent or mortgage?”, (5)”Had services turned off by the gas or electric company, or the oil company wouldn’t deliver oil because payments were not made?”, (6) “Had someone who needed to see a doctor or go to the hospital but didn’t go because you could not afford it?” and (7) “Had someone who needed a dentist but couldn’t go because you could not afford it?” [121,122,123,124,125,126,127]. Subjective financial status predicts health beyond objective SES [121,123,124,128,129,130].

#### 2.3.4. Confounders

Age, sex, ethnicity, and marital status were the confounders. Parents reported adolescents’ age. Age was calculated as the distance of the date of birth to the date of enrollment to the study. Age was measured in years. Sex was a dichotomous variable with males as 1 and females as 0. Parental marital status was a dichotomous variable: Married = 1, unmarried = 0 (reference category). Parents reported their ethnicity. Participants’ ethnicity was coded as 1 for Hispanic and 0 for non-Hispanic.

### 2.4. Data Analysis

We used Statistical Package for the Social Sciences (SPSS) for our data analysis. Frequency (%) and mean (standard deviation [SD]) were described overall and by race. We used Pearson Chi-square and independent samples *t*-test to compare Blacks and White adolescents. To perform our multivariable analyses, we ran four multivariable linear regressions. The independent variable was the SES indicator (parental education). The outcome was left lateral OFC activity during the N-Back task. All these models controlled for age, sex, financial difficulties, and marital status. *Model 1* was performed in Whites. *Model 2* was performed in Blacks. *Model 3* was performed in the pooled sample without the interaction term. *Model 4* was performed in the pooled sample with the interaction term. We reported unstandardized regression coefficients (b), standard errors (SE), 95% confidence interval (CI), *t* value, and their *p*-values. Any *p*-value of less than 0.05 was considered statistically significant. We did not adjust for multiple comparisons because, despite extensive fMRI data in the ABCD study, other brain regions’ available data were not analyzed. As we only analyzed data on lateral OFC function, we kept our *p*-value threshold as 0.05.

### 2.5. Ethics

The study protocol of the ABCD study received approval from the Institutional Review Board (IRB) of the University of California, San Diego. Adolescent participants gave assent. Adult participants (parents) signed informed consent [116]. As our analysis applied fully de-identified data, our study was exempt from a full IRB review by our institution.

## 3. Results

### 3.1. Descriptives

Table 1 shows the descriptive characteristics of the 4290 8–11 years old participating adolescents who were either White (*n* = 3436; 80.1%) or Blacks (*n* = 854; 19.9%). This table also describes the descriptive characteristics of the pooled sample overall and by race. Black and White adolescents differed in family SES but not age or gender or the left lateral OFC activity. Compared to White adolescents, Black adolescents were less likely to be from married families, had lower parental education, and had more financial stress.

### 3.2. Race-Specific Associations

Table 2 reports the results of two race-specific models for the N-Back task results in White and Black children. *Model 1* was performed in White adolescents, and *Model 2* was performed in Black adolescents. We found that parental education was associated with left lateral OFC function during the N-Back task in White but not Black adolescents.

### 3.3. Overall Associations

Table 3 reports the results of regressions overall. In *Model 3*, parental education was not correlated with left lateral OFC activity during the N-Back task. In *Model 4*, a significant interaction was found, suggesting that parental education and left lateral OFC activity during the N-Back task show a stronger positive association in White than Black adolescents.

## 4. Discussion

Parental education was associated with White but not Black OFC activity during the N-Back task. We also found an interaction confirming the same results.

Several studies have explored separate, additive, or multiplicative effects of race and SES on brain function. Most of these studies, however, have investigated separate effects of SES and race. There are only a few, if any, on the multiplicative effects of SES and race on brain development [63,131]. In addition, across SES indicators, the most common indicator has been poverty status, followed by income [15,32,33]. Parental education attainment has not been commonly investigated. Regarding brain regions and structures, most research has studied the amygdala and limbic system [15,32,33], rather than the left lateral OFC. Finally, many scholars do not wish to explore racial differences in brain imaging and function to avoid conflict. This is an overly politicized area of research with significant policy implications. Political correctness has reduced the likelihood of researchers to study how race and SES interact on brain development.

A study explored the associations between family SES (childhood poverty) and functional connectivity between the following brain regions: The hippocampus, amygdala, superior frontal cortex, lingual gyrus, posterior cingulate, and putamen. The study showed that childhood poverty predicts a lower level of connectivity between these regions, and these reduced brain connectivities mediate the effect of childhood poverty on adolescents’ depression [131]. In a series of fMRI publications, a group of researchers, including Javanbakht, established a link between low family SES and functional connectivities between PFC, amygdala, and other brain regions [15,32,33]. These altered connectivities may be why low SES is associated with hyperactivation of the reward network and hypoactivation of the executive network [63]. Thus, the effects of SES and poverty go beyond a particular brain structure and can be seen for connectivity between several brain structures that regulate memory, executive functioning, cognition, and emotion [132]. It is still unknown to what degree the effects of poverty on brain functions are mediated or moderated by positive parenting [133].

Our study findings suggested that Black adolescents face double jeopardy. While race is associated with some altered function of the hippocampus, low SES is also another risk factor for them. There is, however, racial variations in the effects of SES on hippocampus activation during an N-Back task. For Blacks, low SES may come with a higher impact on their hippocampus. The more salient effects of low SES on the hippocampus of Black than White adolescents may be due to the cumulative effects of adversities in the life of racial and ethnic minorities and underserved populations. Racial discrimination and race-related stress may also have some role. Racial discrimination has been shown to impact a wide array of brain regions such as the PFC, anterior insula, putamen, amygdala, caudate, hippocampus, anterior cingulate, and medial frontal gyrus [134].

However, our study is not in support of most previous epidemiological studies that have explored racial differences in the health effects of SES. Many studies have shown more significant effects of SES on outcomes for White than Black adolescents [87]. For example, family SES has shown larger effects on ADHD [90], anxiety [98], aggression [96], tobacco dependence [96], school bonding [135], school performance [83], and overall health [136] for White than Black adolescents. This epidemiological research introduces family SES as a more salient determinant of impulsivity for White than Black adolescents [137]. Thus, we observe poor mental health, physical health, and risk behaviors in high SES Black adolescents [89,90]. These patterns are described as MDRs and hold across age groups, SES indicators, and health outcomes [88].

Differential effects of SES for Black and White families contribute to the transgenerational transmission of inequalities [89,96,136]. Differential effects of SES mean that equal SES generates unequal outcomes for the next generation of adolescents, which means the reproduction of inequalities across generations for Blacks. However, most of the previous studies on MDRs have relied on self-reported outcomes and family SES. Thus, the evidence lacked biological and brain imaging studies that test differential effects of SES on adolescents’ brain function. This paper extended the existing literature by testing such patterns on brain development.

### 4.1. Cautionary Note

In this study, we conceptualized and theorized race as a social factor (a proxy of poverty and SES) on how the brain is affected by low or high SES (parental education). Our approach is different from studies that explore racial variation in brain function or structure as such differences are innate and non-modifiable. We believe that the observed racial differences are more to do with living conditions than genetic predisposition. In this investigation, we studied the former rather than the latter.

### 4.2. Future Research Directions

There is a need for identification and elimination of structural causes of MDRs in Black adolescents and families. Some of the suspects that require future research include racial segregation, school segregation, stress, or exposure to toxins such as air pollutants and lead. These environmental factors may have a role in reducing the health effects of SES for Black families. Labor market discrimination, job availability, discrimination, and segregation may play a role in this regard. There is a need to compare other racial and ethnic groups such as Asian Americans, Native Americans, Hispanics, and immigrants for the effects of SES on the left lateral OFC. Research should also go beyond the left lateral OFC and include other structures that have implications for emotion regulation, memory, cognition, learning, and behaviors.

### 4.3. Limitations

To list the study limitations, one is the cross-sectional design. Due to the design issue, findings should not and cannot be interpreted as causation but rather an association between race, SES, and brain development. SES and brain development have bidirectional associations; thus, future research should also address reverse causation. Second, we only had two SES indicators, namely parental education and financial difficulties. This is particularly important because neighborhood and contextual factors could be why parental education does not generate the same outcome for Black and White families. Third, N-Back provides insight regarding both emotion regulation as well as working memory. This study, however, exclusively focused on a brain mechanism that is involved in working memory.

## 5. Conclusions

In summary, high parental education is correlated with the left lateral OFC activation during the N-Back task in a national sample of White but not Black American adolescents. This observation is also supported by an interaction in the pooled sample suggesting that the magnitude of parental education’s effect on the left lateral OFC function during the N-Back task is less pronounced for Black than White adolescents. More research is needed on the complexities between the effects of race, SES, and social environment on adolescents’ brain development, including but not limited to the left lateral OFC function and other structures with the implication in decision making, learning, and memory.

## Figures and Tables

**Table 1:** Descriptive data overall and by race (*n* = 4290).

	All (*n* = 4290)		Whites (*n* = 3436)		Blacks (*n* = 854)	
	*n*	%	*n*	%	*n*	%
Ethnicity * ^a^						
Non-Hispanic	3582	83.5	2811	81.8	771	90.3
Hispanic	708	16.5	625	18.2	83	9.7
Sex * ^a^						
Male	1985	46.3	1581	46	404	47.3
Female	2305	53.7	1855	54	450	52.7
Family Structure * ^a^						
Not-Married	1258	29.3	707	20.6	551	64.5
Married	3032	70.7	2729	79.4	303	35.5
	**Mean**	**SD**	**Mean**	**SD**	**Mean**	**SD**
Age (Year) * ^b^	9.49	0.51	9.50	0.50	9.49	0.51
Parental Education * ^b^	0.94	0.15	0.96	0.12	0.87	0.20
Subjective Financial Status * ^b^	16.89	2.48	17.22	2.36	15.56	2.53
the left lateral OFC Function * ^b^	0.50	0.86	0.46	0.69	0.66	1.34

* *p* < 0.05 for a comparison of Whites and Blacks. ^a^ Chi-Square test, ^b^ independent samples *t*-test; OFC: Orbito-Frontal Cortex.

**Table 2:** Linear regressions by racial group.

	Model 1 White							Model 2 Black						
	b	SE		95% CI		t	*p*	b	SE		95% CI		t	*p*
Ethnicity	0.04	0.03	0.02	−0.03	0.10	1.11	0.266	−0.06	0.16	−0.01	−0.36	0.25	−0.37	0.708
Sex (Male)	0.06	0.02	0.04	0.01	0.11	2.52	0.012	−0.02	0.09	−0.01	−0.20	0.17	−0.17	0.864
Age	−0.09	0.02	−0.06	−0.13	−0.04	−3.73	<0.001	−0.10	0.09	−0.04	−0.27	0.08	−1.06	0.291
Married	−0.01	0.03	−0.01	−0.07	0.05	−0.43	0.666	−0.14	0.10	−0.05	−0.34	0.07	−1.32	0.187
Subjective Financial Status	−0.06	0.10	−0.01	−0.25	0.14	−0.59	0.557	0.04	0.23	0.01	−0.42	0.49	0.17	0.867
Parental education	0.01	0.01	0.04	0.00	0.02	2.22	0.026	−0.03	0.02	−0.06	−0.07	0.01	−1.62	0.105
Constant	1.10	0.25		0.61	1.59	4.38	<0.001	2.08	0.91		0.29	3.87	2.28	0.023

**Table 3:** Linear regressions overall.

	Model 3 Main Effects							Model 4 Main Effects + Interaction						
	B	SE		95% CI	t	p	*p*	b	SE		95% CI	t	p	*p*
Race (Blacks)	0.04	0.03	0.02	−0.03	0.10	1.11	0.266	0.04	0.03	0.02	−0.03	0.10	1.11	0.266
Ethnicity	−0.01	0.04	0.00	−0.08	0.07	−0.16	0.875	0.02	0.04	0.01	−0.05	0.09	0.51	0.613
Sex (Male)	0.04	0.03	0.02	−0.01	0.09	1.63	0.103	0.04	0.03	0.03	−0.01	0.10	1.68	0.093
Age	−0.09	0.03	−0.05	−0.14	−0.04	−3.54	<0.001	−0.09	0.03	−0.05	−0.14	−0.04	−3.45	0.001
Married	−0.05	0.03	−0.03	−0.12	0.01	−1.64	0.102	−0.05	0.03	−0.02	−0.11	0.02	−1.40	0.162
Subjective Financial Status	−0.01	0.10	0.00	−0.19	0.18	−0.08	0.933	−0.04	0.12	−0.01	−0.29	0.20	−0.35	0.724
Parental education	0.00	0.01	0.00	−0.01	0.01	0.05	0.960	0.01	0.01	0.04	0.00	0.03	1.83	0.067
Race × Subjective Financial Status								0.07	0.19	0.03	−0.31	0.44	0.35	0.729
Race × Parental education								−0.05	0.01	−0.37	−0.08	−0.02	−3.70	<0.001
Constant	1.36	0.27		0.82	1.90	4.94	<0.001	1.14	0.29		0.58	1.71	3.99	<0.001

## Data Availability

Data are available at The National Institute of Mental Health Data Archive (NDA) accessible at https://nda.nih.gov/abcd (accessed on 22 March 2021).
